# Opioid‐Related Challenges Faced by Palliative Healthcare Providers in Both Hospital and Home Care Settings: A Multi‐Center‐Based Descriptive Cross‐Sectional Study

**DOI:** 10.1002/puh2.70013

**Published:** 2025-03-19

**Authors:** Mastura Kashmeeri, A. N. M. Shamsul Islam, Palash Chandra Banik

**Affiliations:** ^1^ Department of Public Health & Hospital Administration NIPSOM Dhaka Bangladesh; ^2^ Department of Noncommunicable Diseases Bangladesh University of Health Sciences (BUHS) Dhaka Bangladesh; ^3^ Center for Higher Studies and Research Bangladesh University of Professionals Dhaka Bangladesh

**Keywords:** addiction, opioid‐related challenges, opioids, pain management, pain policy, palliative care

## Abstract

**Background:**

Palliative care aims to alleviate suffering and improve the quality of life for patients with life‐limiting illnesses through effective pain management with opioids. Despite its global importance, opioid use in palliative care faces significant challenges, particularly in resource‐poor settings like Bangladesh. In Bangladesh, opioid use is notably low, with disparities between urban and rural areas. Cultural stigmas, strict regulations, and inadequate healthcare provider training further obstruct effective pain management.

**Objective:**

This study aims to explore the challenges healthcare providers face regarding opioid use in both hospital and home‐based palliative care settings.

**Method:**

A descriptive cross‐sectional study was conducted using face‐to‐face semistructured interviews with 135 licensed healthcare providers from August to September 2022.

**Results:**

The respondents, predominantly young (57% aged 20–39) and female (68.9%), primarily manage late‐stage cancer patients (98.5%), with pain (100%) being the primary symptom treated. Morphine syrup is commonly used (68.1%), whereas oxycodone and buprenorphine are unavailable. Side effects such as deep sedation (43.7%) and addiction (34.1%) highlight the need for careful monitoring. Limited awareness (10.4%) of national opioid policies results in inconsistent practices (*p* = 0.001) and (*p* = 0.004). Prescribing restrictions (60.7%) and dispensing rights issues contribute to operational challenges, affecting patient access to pain management. Misconceptions about palliative care (32.6%) and inter‐departmental non‐cooperation (38.5%) hinder patient referrals (*p* = 0.001) and continuity of care.

**Conclusion:**

Inconsistent awareness of opioid policies causes varied practices and attitudes. Addressing referral challenges and prescribing restrictions requires interdisciplinary solutions, enhanced education, better policy dissemination, and standardized guidelines for effective palliative care opioid management.

## Graphical abstract

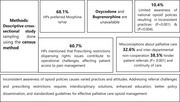

## 1 Background

Palliative care focuses on alleviating suffering and enhancing the quality of life for those with life‐limiting illnesses, with opioid analgesics being a crucial component for pain management. Despite their importance, access to these medications is highly unequal, especially in resource‐constrained settings. According to the World Health Organization, around 5 billion people lack access to proper pain medications, affecting 5.5 million terminal cancer patients and many others with end‐of‐life conditions. Although high‐income countries account for 68% of global medical opioid consumption, lower middle income countries consume only 7% [1, 2]. The disparity is stark, with developed nations like the United States and European countries having established guidelines to ensure opioid availability in palliative care, contrasted with the limited access in countries like Bangladesh, where annual morphine consumption is just 0.05 mg per capita [1, 3, 4, 5, 6]. Barriers in low‐ and middle‐income countries include strict regulations, addiction fears, and insufficient education among healthcare providers about opioid use [6, 7, 8, 9]. The Lancet Commission has added opioids to the essential service package for LMCI countries [10, 11]. Southeast Asia presents a diverse landscape in terms of opioid use in palliative care. Countries like India and Nepal experience considerable challenges due to restrictive regulations, inadequate healthcare provider training, and limited opioid availability. Although India's legal frameworks permit opioid use for pain management, bureaucratic hurdles, and lack of awareness impede effective implementation [1, 12, 13]. Conversely, Singapore and Malaysia have successfully integrated palliative care into their healthcare systems, improving opioid access despite ongoing challenges posed by cultural attitudes toward pain and opioid use [13, 14, 15, 16]. In Bangladesh, access to essential pain medications is notably restricted, especially in rural areas. Stringent regulatory controls hinder the importation and distribution of opioids, and inadequate training in pain management among healthcare providers leads to under‐prescription or inappropriate use [17, 18, 19, 20]. Cultural attitudes, including fears of addiction and misuse, further discourage both prescribing and accepting opioid treatments, resulting in unnecessary suffering [21]. Urban centers like Dhaka have better access to healthcare facilities and medications, including opioids, compared to rural areas. However, regulatory barriers, lack of training, and cultural stigmas persist even in these urban settings. Rural areas face more pronounced difficulties, with healthcare providers often lacking the necessary resources and knowledge to manage pain effectively with opioids [5, 17, 18, 19, 20, 21, 22]. This multi‐center descriptive cross‐sectional study aims to investigate the opioid‐related challenges faced by palliative healthcare providers in both hospital and home care settings across Bangladesh. By comparing findings with global data and studies from Southeast Asia, this study seeks to provide a comprehensive understanding of the issues and suggest potential solutions to improve opioid access and pain management in palliative care settings in Bangladesh.

## 2 Methods

### 2.1 Study Design and Settings, Sample Size, and Criteria

This descriptive cross‐sectional study selected the sample using the census method. A total of 135 participants voluntarily participated out of 148. The remaining 13 were unreachable; 5 were on leave, and 8 refused to provide informed written consent (Table 1). Providers who were physically or mentally unfit or uninvolved were excluded. Data collection took place from August to September 2022. The study included licensed healthcare providers such as doctors, nurses, palliative care assistants (PCAs), ward staff, and PCAs with at least 1 year of experience in palliative care in Dhaka City, Bangladesh. Data were collected from BSMMU, Dhaka Medical College & Hospital, Delta Medical College & Hospital, National Institute of Cancer Research Hospital, and community‐based data were collected from community‐based palliative care projects of BSMMU in collaboration with WHPCA in Korail and Narayanganj City Corporation from Narayanganj. Field data were verified post‐interview.

**TABLE 1:** Distribution according to socio‐demographic traits.

	Frequency	Percentage
**Age range (years)**		
20–39	77	57.0
40–59	55	40.7
Mean age	33.59 ± 8.050	
**Sex**		
Male	42	31.1
Female	93	68.9
**Religion**		
Islam	112	83.0
Hinduism	19	14.1
Christianity	4	3
**Educational qualification**		
Secondary	9	6.7
Higher secondary	15	11.1
Graduation	85	63.0
Post‐graduation	26	19.3
**Occupation**		
Doctor	47	34.8
Nurse	48	35.6
Ward staff	10	7.4
PCA	22	16.3
Pharmacist	8	5.9
**Designation**		
Senior doctors (assistant professor/associate Professor/professor)	5	3.7
Junior doctors (medical officer/resident doctor)	42	31.1
SSN	48	35.6
PCA/ward staff	32	23.7
Pharmacist	8	5.9
**Working experience in the designated post (years)**		
1–3	48	35.6
4–6	50	37.0
7–9	21	15.6
10–above	16	11.9
**Category of hospital serving for**		
Medical college hospital	29	21.5
Specialized hospital	23	17.0
Super specialized hospital	54	40.0
Community‐based treatment center	29	21.5

Abbreviations: PCA, palliative care assistant; SSN, senior staff nurse.

### 2.2 Data Collection Process and Analysis

Pre‐tested, self‐developed semistructured, and self‐reported questionnaires were provided for interviews lasting approximately 30 min. Adjustments were made on the basis of responses, and new options were added during the final analysis. The survey was initially created in English, then translated into Bangla, and analyzed using SPSS software. Categorical and numerical variables were handled separately, with a 5‐point Likert scale used for scoring: “Never” was scored as “0,” “Almost never” as “1,” “Sometimes” as “2,” “Fairly often” as “3,” and “Very often” as “4.” Descriptive statistics were used for qualitative and quantitative variables, whereas inferential statistics were used to identify relationships among the variables.

### 2.3 Ethical Considerations

Ethical approval was obtained from the Institutional Review Board (IRB) of NIPSOM (Memo no: NIPSOM/IRB/2017/09), and the hospital's director granted verbal and written permissions. Participants provided informed consent, and their confidentiality was strictly protected. The investigators ensured privacy and secure data storage, limiting transfer and ensuring no sharing with non‐study personnel.

## 3 Results

The age distribution of respondents indicates that a majority (57%) are aged 20–39, with a significant representation (40.7%) of individuals aged 40–59, balancing youthful perspectives and seasoned experience. The gender distribution shows a female majority (68.9%), reflecting broader trends in healthcare professions. Most respondents are Muslim (83%), aligning with regional demographics. Education‐wise, 63% have completed graduation and 19.3% possess postgraduate degrees (Table 1).

The respondents, representing diverse healthcare professionals in palliative care units, primarily manage late‐stage cancer patients (98.5%). They also care for patients with end‐stage cardiovascular disease (30.4%), stroke and CNS disease (19.3%), and end‐stage chronic kidney disease (28.9%). Common symptoms treated with opioids include pain (100%), breathlessness (61.5%), terminal delirium (55.6%) (Tables S1 and S2). Morphine, in various preparations (tablets, injections, oral solution), is predominantly used, with morphine syrup being the most preferred (68.1%). Oxycodone and buprenorphine are never used. In inpatient clinics, morphine vials are available (68.1%), whereas buprenorphine TTS is not (100%). Tramadol and pethidine are moderately used (Tables 2 and 3).

**TABLE 2:** Which one is your preferred opioid analgesic; Likert scale (0–4).

Opioid analgesic	Never	Rarely	Sometimes	Fairly often	Almost always
Morphine vials	‐	‐	25 (18.5%)	67 (49.6%)	43 (31.9%)
Morphine syrup	‐	‐	13 (9.6%)	30 (22.2 %)	92 (68.1%)
Morphine SR tablets	‐	3 (2.2%)	24 (17.7%)	25 (18.5%)	83 (61.5%)
Morphine IR tablets	‐	‐	21 (15.6%)	61 (45.2%)	53 (39.3%)
Oxycodone vials	135 (100%)	‐	‐	‐	‐
Oxycodone SR tablets	135 (100%)	‐	‐	‐	‐
Fentanyl vials	‐	‐	90 (66.7%)	36 (26.7%)	9 (6.7%)
Buprenorphine TTS	135 (100%)	‐	‐	‐	‐
Pethidine	6 (4.4%)	18 (13.3%)	83 (61.5%)	25 (18.5%)	3 (2.2%)
Methadone	135 (100%)	‐	‐	‐	‐
Tramadol	12 (8.9%)	27 (20%)	61 (45.2%)	35 (25.9%)	‐

Abbreviations: IR, immediate release; SR, sustained release; TTS, transdermal therapeutic system.

**TABLE 3:** Distribution of respondents as strong opioids available in IPD (*n* = 135).

Opioid analgesic	Never	Rarely	Every once in a while	Sometimes	Almost always
Morphine vials	‐	‐	13 (9.6%)	92 (68.1%)	30 (22.2%)
Morphine syrup	‐	42 (31.1%)	33 (24.4%)	39 (28.9%)	21 (21%)
Morphine SR tablets	‐	3 (2.2%)	24 (17.7%)	25 (18.5%)	83 (61.5%)
Morphine IR tablets	‐	‐	27 (20%)	61 (45.2%)	53 (39.3%)
Fentanyl vials	107 (79.3%)	28 (20.7 %)	‐	‐	‐
Buprenorphine TTS	135 (100%)	‐	‐	‐	‐
Pethidine	‐	69 (51.1%)	19 (14.1%)	39 (28.9%)	8 (5.9%)
Tramadol	12 (8.9%)	27 (20%)	61 (45.2%)	21 (15.6%)	9 (6.7%)

Abbreviations: IPD, inpatient department; IR, immediate release; SR, sustained release; TTS, transdermal therapeutic system.

Reported opioid side effects include deep sedation (43.7%), respiratory depression (34.1%), vomiting (25.2%), and tolerance (28.9%). Concerns about opioid addiction (23%) and dependence (14.1%) highlight the need for stringent prescribing guidelines and patient education (Table S3).

Identifying opioid addiction in patients (34.1%) is a significant concern, along with addiction among caregivers (5.9%) and healthcare providers (7.4%) (Table S4). Most respondents (80.7%) reported strict supervision of opioid storage, with 34.1% collecting empty vials, bottles, and strips. All respondents agreed that opioid dispensing rights are restricted, with senior staff nurses (SSNs) (69.6%), pharmacists (20%), and PCAs (10.4%) dispensing opioids (Table 4).

**TABLE 4:** Opinion about the handling of opioids (*n* = 135).

	Frequency (*f*)	Percentage
**Storing capacity**		
Strict supervision	109	80.7
Empty vial/bottle/strep collection	46	34.1
Opioid dispense rights restricted for		
SSN	94	69.6
Pharmacist	27	20.0
PCA	14	10.4
**Restriction on opioid prescriptions is necessary**		
No	17	12.6
Yes	82	60.7
I don't know	25	18.5
Undecided	11	8.1
**Opioid prescribing rights restricted for**		
MO	79	58.5
Assistant professor	39	28.9
Associate professor/professor	17	12.6

Abbreviations: PCA, palliative care assistant; SSN, senior staff nurse.

Awareness and implementation of national opioid policies and guidelines vary, with only 10.4% aware of existing guidelines, whereas 37.8% are unaware, and 24.4% are undecided (Table 5).

**TABLE 5:** Distribution of respondents as opioid‐related policies, guidelines and referral of patients (*n* = 135).

	Frequency (*f*)	Percentage
**National policies on opioid use**		
Has own management policies	33	24.4
I don't know	45	33.3
Undecided	57	42.2
**National guideline on opioid use**		
Yes	14	10.4
I don't know	51	37.8
Undecided	33	24.4
Not published yet	37	27.4
**Challenges on patient referral to another department**		
Easy, systematic, and friendly	10	7.4
Misconception about PC	44	32.6
Non‐cooperation	52	38.5
Lack of knowledge about PC settings	29	21.5

Challenges in patient referral, including misconceptions about palliative care (32.6%), non‐cooperation (38.5%), and lack of knowledge about palliative care settings (21.5%), suggest systemic issues hindering effective patient management. Most respondents (78.5%) mentioned obtaining morphine from local private pharmaceutical companies (Table S5).

Statistical analysis revealed significant differences in attitudes, prescribing forms (Table 6), and working knowledge based on awareness of national opioid policies and guidelines (Table 7), as well as challenges in patient referral. However, there were no significant differences in the inpatient department (IPD) concerning opioid prescription restrictions and national policies. Significant *p* values were shown in Tables 6 and 7.

**TABLE 6:** Association between prescribing practice and opioid‐related challenges at different levels (*n* = 135).

	Overall attitudes			Overall prescribing form			Overall working knowledge			Overall impending IPD		
*N*	Mean	SD	*N*	Mean	SD	*N*	Mean	SD	*N*	Mean	SD
**Opioid prescription restriction**												
No	17	2.12	0.485	17	1.94	0.243	17	2.06	0.243	17	2.00	0.17
Yes	82	1.95	0.469	82	1.74	0.517	82	2.02	0.517	82	2.01	0.07
I don't know	25	2.08	0.493	25	1.56	0.507	25	1.92	0.507	25	2.12	0.12
Undecided	11	1.91	0.539	11	1.91	0.302	11	1.91	0.302	11	2.55	0.15
***p* value**	0.409			0.051			0.707			0.086		
**Opioid should be prescribed**												
MO/residents	79	2.00	0.48	79	1.76		79	1.97	0.48	79	2.08	0.50
Assistant professor	39	1.95	0.51	39	1.69		39	2.10	0.50	39	2.10	0.69
Associate professor and professors	17	2.06	0.42	17	1.82		17	1.88	0.50	17	2.00	0.64
***p* value**	0.720			0.618			0.255			0.870		
**Opioid should be dispensed**												
SSN	94	1.99	0.475	94	1.78	0.490	94	2.03	0.538	94	1.98	0.672
Pharmacist	27	2.04	0.518	27	1.59	0.501	27	1.93	0.474	27	2.19	0.622
PCA	14	1.93	0.475	14	1.86	0.363	14	1.93	0.267	14	2.50	0.519
***p* value**	0.788			0.148			0.514			0.14		
**Availability of national policy related to opioids**												
I don't know	45	2.09	0.288	45	1.56	0.503	45	1.75	0.484	45	2.18	0.716
Undecided	57	2.05	0.225	57	1.82	0.384	57	1.69	0.468	57	2.00	0.655
Have own management policy	33	1.76	0.830	33	1.88	0.545	33	1.95	0.225	33	2.06	0.609
***p* value**	0.004			0.004			0.001			0.407		
**Availability of national guidelines related to opioids**												
Yes	14	2.00	0.001	14	1.71	2.00	14	1.93	0.730	14	1.21	0.426
I don't know	51	2.00	0.346	51	1.73	1.84	51	1.65	0.483	51	1.53	0.504
Undecided	33	1.79	0.600	33	1.76	2.09	33	2.12	0.545	33	1.30	0.467
Has not published	37	2.16	0.553	37	1.78	2.14	37	2.68	0.665	37	1.76	0.435
***p* value**	0.013			0.943			0.032			0.001		
**Challenges on patient referral to another department**												
Easy, systematic and friendly	10	2.00	0.001	10	2.00	0.001	10	1.00	0.001	10	2.00	0.001
Misconception about PC	44	1.98	0.505	44	1.66	0.479	44	2.00	0.571	44	2.02	0.762
Non‐cooperation	52	2.06	0.461	52	1.79	0.412	52	2.13	0.345	52	1.98	0.610
Lack of knowledge about PC settings	29	1.90	0.557	29	1.72	0.649	29	2.10	0.310	29	2.34	0.670
***p* value**	**0.545**			**0.202**			**0.001**			**0.010**		

*Note*: **Overall attitudes:** Opioid related national policies (*p* = 0.004), Opioid related national guidelines (*p* = 0.013); **Overall prescribing forms:** Opioid related national policies (*p* = 0.004); Overall working knowledge: Opioid related national policies (*p* = 0.001), Opioid related national guidelines (*p* = 0.032), Referral challenges (*p* = 0.001); Overall impending IPD: Opioid related national guidelines (*p* = 0.001), Referral challenges (*p* = 0.010).

Abbreviations: PCA, palliative care assistant; SSN, senior staff nurse.

**TABLE 7:** Association between physical pain service and opioid‐related challenges at different levels (*n* = 135).

	Satisfactory pain management			Satisfactory side effect management		
**Opioid prescription restriction**						
No	17	1.53	0.514	17	1.53	0.514
Yes	82	1.60	0.606	82	1.49	0.503
Undecided	25	1.72	0.6.14	25	1.56	0.507
I don't know	11	1.73	0.467	11	1.45	0.522
***p* value**	0.657			0.910		
**Opioid should be prescribed by**						
MO/Residents	79	1.56	0.549	79	1.56	0.500
Assistant professor	39	1.69	0.614	39	1.69	0.505
Associate professor and professors	17	1.76	0.664	17	1.76	0.493
***p* value**	0.280			0.261		
**Opioid should be dispensed by**						
SSN	94	1.59	0.594	94	1.49	0.503
Pharmacist	27	1.70	0.609	27	1.56	0.506
PCA	14	1.71	0.469	14	1.50	0.519
***p* value**	0.538			0.835		
**Availability of national policy related to opioids**						
I don't know	45	1.73	0.618	45	1.58	0.499
Undecided	57	1.60	0.593	57	1.44	0.501
Have own management policy	33	1.52	0.508	33	1.52	0.508
***p* value**	0.242			0.379		
**Availability of national guidelines related to opioids**						
Yes	14	1.21	0.426	14	1.21	0.426
I don't know	51	1.57	0.608	51	1.53	0.504
Undecided	33	1.76	0.663	33	1.30	0.467
It has not been published yet	37	1.73	0.450	37	1.76	0.435
***p* value**	0.015			0.001		
**Challenges on patient referral to another department**						
Easy, systematic, and friendly	10	1.50	0.527	10	1.50	0.527
Misconception about PC	44	1.61	0.655	44	1.61	0.493
Non‐cooperation	52	1.62	0.599	52	1.48	0.505
Lack of knowledge about PC settings	29	1.69	0.471	29	1.38	0.494
***p* value**	0.843			0.263		

Abbreviations: PCA, palliative care assistant; SSN, senior staff nurse.

## 4 Discussion

The study indicates that 57% of healthcare professionals are aged 20–39 years, and 40.7% are aged 40–59 years. This aligns with findings from studies like those by McPherson et al., which suggest that younger healthcare professionals often receive more recent training in modern medical education and opioid management protocols [22].

A recent study on young health professionals in Dhaka revealed that their understanding of palliative care is basic but often incomplete, highlighting concerns about opioid use for pain management and the regulatory restrictions and lack of training on opioid use [20]. A balanced mix of young and experienced professionals is essential for effectively addressing complex issues related to opioids. By promoting better understanding and fostering more positive attitudes, young health professionals can play a crucial role in advancing palliative care and addressing opioid‐related challenges in Bangladesh.

The significant percentage of female respondents (68.9) reflects the broader trend in healthcare, where women predominantly fill nursing and allied health roles. Studies such as those conducted by Buchan et al. highlight the gendered nature of certain healthcare roles, especially nursing [23]. Ferrell's research discusses the significant role of female health professionals, particularly in oncology nursing, in providing compassionate and holistic care to cancer patients. Women health professionals often excel in offering empathetic support and understanding patients’ and their families’ complex emotional and physical needs [24]. Their contribution to compassionate and effective care can be enhanced by advocating for education and training for oncology nurses to improve their knowledge and skills in pain management, including opioid pharmacology and symptom control.

The majority of Muslim respondents (83%) in Bangladesh match the regional demographics, impacting cultural and ethical viewpoints on pain management. Payne et al.’s research discusses how religious beliefs affect healthcare practices and patient care policies [25]. Cultural beliefs in Bangladesh may influence attitudes toward pain management and opioid use. Some cultural beliefs view pain as a natural part of life or prioritize non‐pharmacological treatments over opioid medications, significantly influencing end‐of‐life care decisions [5, 13].

Religious teachings and ethical considerations may impact physicians’ willingness to prescribe opioids, particularly due to concerns about addiction and misuse. A similar situation exists in Vietnam, where certain cultural beliefs may perceive pain as a natural part of life or emphasize enduring suffering without complaint, affecting the acceptance and use of opioid medications [21]. Addressing these issues involves enhancing infrastructure, training healthcare providers in pain management and palliative care, and dispelling stigma and misconceptions about opioid use through public awareness campaigns [11, 14]. This underscores the crucial role of cultural sensitivity and well‐rounded policy approaches in tackling religious and cultural influences on opioid‐related challenges to enhance global outcomes in pain management and palliative care.

The survey revealed that more than two‐thirds of respondents had completed their graduation, whereas 19.3% possessed post‐graduate degrees. Palliative care is less popular in Bangladesh, and there is a notable variation in the level of knowledge among physicians, often linked to disparities in their educational backgrounds and training [11, 19]. Physicians who receive more comprehensive education in palliative care are better equipped to manage pain using opioids, though their numbers remain negligible [5, 18, 19, 20]. Research by Knaul et al. indicates that higher education levels among healthcare professionals are linked to better knowledge and practices in palliative care [9]. The survey showed that 98.5% of respondents work with late‐stage cancer patients, indicating the crucial role of opioids in palliative care.

Pain was reported by all respondents (100%) as the most common symptom, emphasizing the vital role of opioids in managing pain [11]. This reflects the global trend in palliative care, where opioids are essential for addressing severe pain in terminal illnesses. Other significant symptoms such as breathlessness, delirium are commonly treated in advanced diseases and these findings are consistent with studies by Clark et al. and Ferrell et al., emphasizing the importance of addressing multiple symptoms in palliative care, not just pain relief [2, 24]. By enhancing medical education through integrating comprehensive palliative care and pain management training into medical school curricula and continuing professional development programs, we could bridge the knowledge gap and improve physicians’ ability to manage pain effectively using opioids.

According to the study, morphine is the most commonly used opioid analgesic due to its affordability and effectiveness, whereas oxycodone, buprenorphine TTS, and methadone are never used here and tramadol and pethidine are used moderately. This trend is consistent with other low‐ and middle‐income countries. The WHO emphasizes morphine as the gold standard for moderate‐to‐severe pain in palliative care settings [5, 26, 27]. In higher‐income countries, morphine is a mainstay in pain management, often supplemented by other opioids like oxycodone and fentanyl. Pethidine is less favored globally due to its neurotoxic metabolite. Conversely, oxycodone is frequently prescribed in the United States for acute and chronic pain, and buprenorphine is widely used in opioid substitution therapy for addiction treatment [28, 29, 30].

In Bangladesh, morphine is available in three main forms: tablets, injections, and oral solutions. Most physicians have poor knowledge and attitudes toward opioid availability and use except oncology physicians [5, 12, 27, 31]. Patients may face obstacles in accessing opioids, including reluctance to prescribe, lack of knowledge among healthcare professionals, restrictive procedures, and high costs. Moreover, side effects of opioids in Bangladesh include constipation, sedation, vomiting, fatigue, occasionally respiratory depression, and tolerance that are consistent with global concerns about the negative impact of these medications [4, 5, 27, 31]. Similarly, in other LMCIs, these opioids are less commonly used due to similar barriers. For instance, a study in Africa highlighted the limited availability and high cost of oxycodone and buprenorphine as significant challenges to their use in clinical practice [32, 33, 34]. Studies have shown similar concerns about opioid‐related adverse effects in the United States and internationally. The high rate of opioid addiction in patients reflects the widespread opioid crisis and challenges in managing chronic pain without promoting dependency [26, 35, 36]. Similar concerns exist among caregivers and healthcare providers in Bangladesh. This highlights the need for careful monitoring and dose management. The discussion emphasizes the importance of managing these medications safely and the need for a balanced approach to effective pain management while preventing opioid misuse and diversion.

In Bangladesh, there are no restrictions on the amount or time limit for prescribing opioids for medical use. However, the main barrier is the restriction on the level of physicians who can prescribe opioids, requiring proper licensing. Complex laws can hinder governments from preventing drug misuse while ensuring a continuous and adequate supply of drugs for legitimate medical purposes [5, 37, 38, 39, 40]. Strict supervision of opioid storage and dispensing, with 80.7% reporting stringent measures, mirrors practices in other countries. The WHO advocates for strict control measures, similar to those in Bangladesh, to ensure the safe use of opioids [1, 5, 37].

The Controlled Drugs Regulations in the United Kingdom and the tiered approach to prescribing rights in countries like Canada reflect this emphasis on rigorous control to prevent misuse [28, 30, 41, 42, 43, 44]. This underscores the importance of fostering interdisciplinary collaboration to optimize opioid prescribing practices and ensure effective pain management across medical specialties. The study highlights significant gaps in the awareness and implementation of national policies and guidelines on opioid use in Bangladesh, with only 10.4% of respondents aware of existing guidelines. Similar challenges are noted in Latin America, where a lack of awareness and inconsistent policy implementation hinder effective pain management [45]. Research by Biswas, Banik, and Ahmad also reveals limited knowledge among Bangladeshi physicians about palliative care, suggesting a need for better dissemination and training [18, 19]. Education and training are crucial to address this, as seen in Asia, where initiatives have improved adherence to guidelines.

The challenges in patient referral, such as misconceptions about palliative care (32.6%) and non‐cooperation (38.5%), require comprehensive strategies to improve interdepartmental collaboration. Studies emphasize the importance of interdisciplinary teamwork in palliative care, as seen in Leclerc et al.’s work and similar issues in Vietnam [21, 46]. Brenneis and Bruera's research underscores the need for interdisciplinary training and clear communication pathways. In Canada, integrated care models facilitate smooth transitions between departments, improving patient management and satisfaction [44]. The necessity for education and training in palliative care is further supported by studies like De Lima et al., which highlight the positive impact of targeted training programs on healthcare professionals’ knowledge and attitudes toward palliative care [47].

### 4.1 Recommendations

To address the complexities of opioid‐related practices in Bangladesh, it is recommended to enhance interdisciplinary collaboration and education. By fostering partnerships among younger healthcare professionals with modern training and experienced practitioners, a balanced approach to opioid management can be achieved. This collaboration should prioritize ongoing education on opioid use and pain management protocols to ensure comprehensive care across all medical specialties.

### 4.2 Limitations

A key limitation of the study lies in the reliance on self‐reported data, which may lead to biases, particularly concerning healthcare professionals' knowledge and attitudes toward opioid use and palliative care. The study lacks detail on training disparities among different age groups and their impact on clinical practice. It also does not consider potential regional differences in Bangladesh, which could affect access to opioids and cultural attitudes toward pain management. Additionally, it focuses narrowly on specific opioids (morphine), without exploring alternative pain management strategies, as it is accepted in Bangladesh. Finally, the study predominantly includes those working with late‐stage cancer patients, potentially limiting the generalizability of the findings across different medical disciplines.

## 5 Conclusion

In conclusion, the socio‐demographic characteristics of healthcare professionals in Bangladesh play a pivotal role in shaping attitudes toward opioid prescribing and palliative care practices. The predominance of younger professionals underscores the potential for innovative approaches to opioid management, given adequate support and training. Addressing gaps in policy awareness and promoting interdisciplinary collaboration are crucial steps toward enhancing opioid safety and efficacy in pain management across the healthcare system.

## Author Contributions

Conception and development of idea: M.K. and S.I. Data collection: M.K. Data analysis: M.K., P.C.B. Writing: M.K. Review and editing: M.K., P.C.B., and S.I.

## Disclosure

The funders had no involvement in the study design, data collection and analysis, the decision to publish, or the preparation of the manuscript. Grammarly and Chat GPT (free version) were used for grammatical correction.

## Conflicts of Interest

The authors declare no conflicts of interest.

## Supporting information



Supporting information

## Data Availability

All data relevant to the study are accessible in Mendeley data DOI:10.17632/b6fyy2zp9h.1 https://data.mendeley.com/preview/b6fyy2zp9h?a=d310d80d‐cb11‐4533‐b407‐6a7fa09ddcbc
