# Supplementary material for: Opioid‐Related Challenges Faced by Palliative Healthcare Providers in Both Hospital and Home Care Settings: A Multi‐Center‐Based Descriptive Cross‐Sectional Study
Source: Public Health Chall. 2025 Mar 19;4(1):e70013. doi: 10.1002/puh2.70013 (PMC12039347; doi:10.1002/puh2.70013)
Supplement: Supplementary file 1 — Supporting information [file PUH2-4-e70013-s001.docx]

***Table S1: Distribution of respondents as per mostly handled patients (n=135)***

| **Name of diagnosis** | **Frequency (f)** | **Percentages (%)** |
| --- | --- | --- |
| Late-stage Cancer | 133 | 98.5 |
| End stage CVD | 41 | 30.4 |
| Stroke & CNSD | 26 | 19.3 |
| End stage CKD | 39 | 28.9 |
| Geriatric | 59 | 43.7 |
| CP | 24 | 17.8 |

***Table S2: Distribution of respondents as per predominant symptom (n=135)***

| **Symptoms** | **Frequency (f)** | **Percentages (%)** |
| --- | --- | --- |
| Pain | 135 | 100 |
| Fatigue | 44 | 32.6 |
| Breathlessness | 83 | 61.5 |
| Disorientation | 75 | 55.6 |
| Constipation | 68 | 50.4 |
| Hemorrhage | 20 | 14.8 |
| Wound | 25 | 18.5 |

***Table S3. Distribution of respondents as predominant side effect of opioid seen in patients (n=135)***

| **Side effect** | **Frequency (f)** | **Percentages (%)** |
| --- | --- | --- |
| Vomiting | 34 | 25.2 |
| Respiratory depression | 46 | 34.1 |
| Deep Sedation or drowsiness | 59 | 43.7 |
| Tolerance | 39 | 28.9 |
| Dependence | 19 | 14.1 |
| Addiction | 31 | 23 |

***S4: Distribution of respondents as opioid addiction prominently seen in patients (n=135)***

| **Side effect** | **Frequency (f)** | **Percentages (%)** |
| --- | --- | --- |
| Patient | 46 | 34.1 |
| Care giver | 8 | 5.9 |
| Health care providers | 10 | 7.4 |

***S5:Distribution of obtaining morphine*** *(****n=135)***

| **Side effect** | **Frequency (f)** | **Percentages (%)** |
| --- | --- | --- |
| Purchase from local market | 106 | 78.58 |
| Available in TC | 21 | 15.51 |
| I don’t know | 8 | 5.91 |

*TC Treatment center*
